# Diet, tooth loss, and dental calculus in Guizhou adults (2024–2025)

**DOI:** 10.3389/fpubh.2026.1829730

**Published:** 2026-07-29

**Authors:** Wei Liu, Deyong Li, Guangliang Yang, Shouyan Gan, Liu Zhang, Jing Yang

**Affiliations:** 1 Department of Stomatology, Liwan Central Hospital of Guangzhou, Guangzhou, Guangdong Province, China; 2 Department of Stomatology, Huishui People's Hospital, Huishui, Guizhou Province, China; 3 Department of Prosthodontics, Guiyang Stomatological Hospital, Guiyang, Guizhou Province, China

**Keywords:** dental calculus, dietary structure, Huishui area, oral health, risk factor, tooth loss

## Abstract

**Objective:**

This study aimed to explore the correlation between dietary structure characteristics and the prevalence of tooth loss and dental calculus in adults residing in Huishui Area of Guizhou Province.

**Methods:**

A total of 4,216 permanent adults aged ≥18 years were recruited using multi-stage stratified random sampling. Face-to-face questionnaire surveys collected dietary intake information, and standardized oral clinical examinations recorded tooth loss and dental calculus grading. Binary logistic regression (for tooth loss) and ordinal logistic regression (for dental calculus severity) were applied, adjusting for age, sex, education, income, brushing frequency, flossing use, smoking and alcohol consumption.

**Results:**

Tooth loss rate was 31.45% (mean 1.7 teeth/person); dental calculus detection rate was 71.00% (Grade I: 34.00%, Grade II: 24.00%, Grade III: 12.00%). Multivariate analysis identified weekly meat intake (OR = 1.20, 95%CI: 1.02–1.42), weekly nut intake (OR = 0.61, 95%CI: 0.46–0.82), daily dairy intake (OR = 0.62, 95%CI: 0.46–0.83), weekly fruit intake frequency (OR = 0.60, 95%CI: 0.44–0.81), weekly vegetable intake frequency (OR = 2.80, 95%CI: 2.00–3.91), and daily vegetable weight (OR = 0.68, 95%CI: 0.53–0.88) as independent factors for tooth loss (all *p* < 0.05). For dental calculus severity, weekly meat intake (cumulative OR = 1.06, 95%CI: 1.02–1.10), daily dairy intake (cumulative OR = 0.89, 95%CI: 0.86–0.93), weekly fruit frequency (cumulative OR = 0.94, 95%CI: 0.90–0.97), weekly vegetable frequency (cumulative OR = 1.05, 95%CI: 1.01–1.10), and daily vegetable weight (≥500 g vs. <150 g: cumulative OR = 0.80, 95%CI: 0.74–0.86) were significant (all *p* < 0.05).

**Conclusion:**

Unbalanced dietary structure is a key modifiable risk factor. The “3-4-500” dietary model provides an evidence-based framework for oral health promotion in underserved populations.

## 1 Introduction

Tooth loss is one of the most common oral disorders in adults, which not only leads to a decline in masticatory and digestive functions but also increases the risk of developing systemic diseases such as cardiovascular and cerebrovascular diseases and Alzheimer’s disease, ultimately impairing quality of life (1, 2). As a primary etiological factor of periodontal disease, dental calculus deposition can induce gingival inflammation and periodontal pocket formation; if left untreated, it can progress to severe periodontitis, further causing tooth loosening and even loss (3). In addition, accumulating evidence has demonstrated a close association between dental calculus deposition and several systemic disorders (4). Huishui Area is located in Qiannan Buyi and Miao Autonomous Prefecture of Guizhou Province, where the Buyi and Miao ethnic groups are the predominant populations. The dietary structure of local residents is characterized by distinct regional and ethnic features, which may have a potential impact on oral health. Dietary habits are well-recognized as important determinants of oral health, yet the specific correlation between dietary structure and the prevalence of tooth loss and dental calculus in this region remains largely unexplored (5, 6).

In the present study, an epidemiological investigation combined with oral clinical examinations was conducted among adults in Huishui Area to analyze the characteristics of local dietary structure and explore the effects of intake frequency and quantity of different foods on tooth loss and dental calculus. The findings of this study are expected to provide data support and practical references for formulating targeted dietary prevention strategies for oral diseases and improving the oral health status of the population in this ethnic minority-concentrated region.

Understanding the relationship between dietary patterns and oral health in diverse populations is essential for developing globally relevant oral health promotion strategies. As part of the broader effort to address oral health disparities in underserved communities worldwide, this study provides unique insights from a Chinese ethnic minority region, contributing to the international evidence base on diet-oral health associations (7, 8).

## 2 Subjects and methods

### 2.1 Study design and sampling

This study was conducted from 1 January 2024 to 31 December 2025.

#### 2.1.1 Sample size calculation

The sample size was calculated using the formula for enumeration data: *N* = *t*^2^pq/*d*^2^ (where *N* = sample size; *p* = estimated overall prevalence rate; *q* = 1 − *p*; *t* = test statistic; *d* = allowable sampling error). Based on domestic epidemiological data, the prevalence of dental calculus in Chinese adults is approximately 88%, and the prevalence of tooth loss in the older adults population is over 87.5% (9). With a significance level of *α* = 0.05 (*t* = 1.96) and an allowable sampling error of *d* = 0.01, the calculated sample size for dental calculus research was 4,056, and for tooth loss research was 4,201. A total of 4,216 subjects were finally enrolled in this study to ensure sufficient statistical power.

#### 2.1.2 Sampling method

A multi-stage stratified random sampling method was adopted. Taking the entire Huishui Area as the research population, 5 towns (sub-district offices) were first randomly selected from 8 towns and 2 sub-district offices according to local population distribution and administrative divisions. Subsequently, 5 villages (streets) were randomly selected from each selected town (sub-district office), resulting in a total of 25 sampling units. Permanent adults aged 18 years and above were recruited from each sampling unit, and a total of 4,216 subjects were included in the final analysis.

### 2.2 Ethics statement

This study was conducted in accordance with the Declaration of Helsinki. The protocol was approved by the Institutional Review Board of Huishui People’s Hospital (Approval No. HY202305, approved on 6 August 2023). All participants were informed of the study aims, procedures, potential risks, and their right to withdraw at any time without consequences. Written informed consent was obtained from all participants prior to enrollment. For illiterate participants, consent was obtained via a witnessed oral consent process, with the witness signing on their behalf.

### 2.3 Data collection

#### 2.3.1 Questionnaire survey

Face-to-face questionnaire surveys were conducted by well-trained oral medical professionals. The questionnaire collected detailed dietary intake information, including the number of days of weekly meat intake, weekly nut intake, daily dairy product consumption, weekly fruit intake frequency, weekly vegetable intake frequency, daily vegetable intake weight, and commonly consumed vegetable types. The questionnaire was adapted from a validated food frequency questionnaire (FFQ) used in a similar rural Chinese population (10). All investigators were uniformly trained to ensure accuracy and consistency.

Daily vegetable intake weight was estimated by trained interviewers using visual aids (standard portion size photographs and household measures, e.g., “one bowl ≈ 150 g,” “one handful ≈ 100 g”). Participants were not required to actually weigh their vegetables; instead, they reported their usual consumption in terms of bowls, plates, or handfuls, which were then converted into grams based on pre-tested conversion tables for locally common vegetables. The same procedure has been used in similar rural Chinese dietary surveys.

Dairy intake referred primarily to liquid milk (cow’s milk) and yogurt. Only liquid milk and yogurt were assessed in this study; solid or semi-solid dairy products (e.g., cheese) were rarely consumed in the study population and thus were not separately quantified. Intake was recorded in milliliters (mL) based on the volume of liquid milk or yogurt consumed. For participants who consumed both, the total volume was summed.

The questionnaire did not distinguish subtypes of meat (e.g., red vs. white, processed vs. unprocessed), fruit (whole vs. juice), or nuts (raw vs. salted/oil-roasted) due to the limited scope of the survey. Thus, the dietary variables represent aggregated intake across all subtypes within each food group. This limitation is addressed further in the discussion.

#### 2.3.2 Oral clinical examination

Standardized oral clinical examinations were performed by two qualified licensed dentists using routine oral examination instruments (mouth mirrors, periodontal probes). All examinations were performed at temporary dental clinics set up in each selected village’s community health service center. Participants were invited to attend by local village doctors and community coordinators. For participants who were bedridden or had severe mobility limitations, home visits were conducted by the same examiners using portable dental mirrors and probes. In both settings, examinations were performed under adequate natural or artificial light with the participant seated in an upright position. The number of missing teeth and the grading of dental calculus were recorded for each subject. All examination results were double-checked and entered into a standardized database. Inter-examiner reliability was assessed on 100 randomly selected participants (10% of the sample). The weighted kappa coefficient for dental calculus grading was 0.84 (95% CI: 0.78–0.90), indicating excellent agreement. For tooth loss count, the intraclass correlation coefficient (ICC) was 0.92.

### 2.4 Diagnostic criteria

Dental calculus: The diagnosis was based on the Simplified Calculus Index (CI-S) recommended in the Group Standard for Basic Periodontal Examination and Evaluation Norms (2020) issued by the Chinese Stomatological Association (11): Grade 0 = no supragingival or subgingival calculus; Grade 1 = supragingival calculus covering less than 1/3 of the tooth surface; Grade 2 = supragingival calculus covering 1/3 to 2/3 of the tooth surface or scattered subgingival calculus at the tooth neck; Grade 3 = supragingival calculus covering 2/3 or more of the tooth surface or continuous and thick subgingival calculus at the tooth neck.

Tooth loss: The diagnosis was in accordance with the Clinical Guidelines for the Diagnosis and Treatment of Dental Defect, Dentition Defect and Edentulism (2022) issued by the National Health Commission of China (12). The total number of missing teeth and the mean number of missing teeth per person were calculated for all subjects. Teeth lost due to trauma, orthodontic extraction, or congenital absence were excluded from the analysis.

### 2.5 Inclusion and exclusion criteria

Inclusion criteria: (1) Long-term residence in Huishui Area for more than 1 year; (2) Aged 18 years and above; (3) Holding Chinese nationality; (4) Voluntarily participating in this study and providing written informed consent.

Exclusion criteria: (1) Tooth loss caused by tumors, trauma, developmental disorders or other non-dietary factors; (2) Complicated with diagnosed severe systemic diseases (e.g., severe cardiovascular and cerebrovascular diseases, poorly controlled diabetes mellitus) based on self-reported medical history or documented medical records. Participants with undiagnosed or suspected systemic conditions were not excluded; only those with a confirmed diagnosis were excluded; (3) Suffering from mental disorders or cognitive impairment, unable to provide accurate dietary and personal information; (4) Refusing to cooperate with oral clinical examinations or questionnaire surveys.

### 2.6 Statistical analysis

All statistical analyses were performed using SPSS 26.0 (IBM Corp., Armonk, NY, USA). Measurement data were expressed as mean ± standard deviation (x̄ ± s). Enumeration data were expressed as rates or percentages (%), and inter-group comparisons were performed using Pearson’s *χ*^2^ test.

Tooth loss analysis: The dependent variable was tooth loss (0 = no tooth loss, 1 = any tooth loss). Binary logistic regression was used to identify independent dietary factors associated with tooth loss. Results are presented as odds ratios (OR) with 95% confidence intervals (CI).

Dental calculus analysis: The dependent variable was dental calculus severity (ordinal: 0 = clean, 1 = Grade I, 2 = Grade II, 3 = Grade III). Ordinal logistic regression (cumulative logit model) was used to assess the association between dietary factors and calculus severity. Results are presented as cumulative ORs with 95% CIs.

Both models were adjusted for the following potential confounders: age (continuous), sex (male/female), education level (ordinal: 1 = primary or below, 2 = middle school, 3 = high school, 4 = college or above), monthly income (ordinal: 1 = ≤2,999元, 2 = 3,000–8,000元, 3 = ≥8,000元), tooth brushing frequency (0 = ≤1 time/day, 1 = ≥2 times/day), flossing use (0 = no, 1 = yes), smoking (0 = no, 1 = yes), and alcohol consumption (0 = no, 1 = yes). Dietary variables were treated as ordinal categories as follows:

Weekly intake (meat, nuts, fruit, vegetables): 0 = never, 1 = 1–2 days/week, 2 = 3–4 days/week, 3 = 5–7 days/week.

Daily dairy intake: 0 = never, 1 = 250 mL, 2 = 500 mL, 3 = ≥750 mL.

Daily vegetable weight: 0 = <150 g, 1 = 150–249 g, 2 = 250–500 g, 3= >500 g.

A two-tailed *p* value < 0.05 was considered statistically significant. Subgroups with sample size <10 were flagged with “#” in the tables to indicate unstable estimates.

## 3 Results

### 3.1 Inter-examiner reliability

The inter-examiner reliability was excellent: the weighted kappa coefficient for dental calculus grading was 0.84 (95% CI: 0.78–0.90), and the intraclass correlation coefficient (ICC) for tooth loss count was 0.92.

### 3.2 Participant characteristics

A total of 4,216 adults were included. The mean age was 45.2 ± 15.6 years (range 18–92). Table 1 summarizes the demographic and oral health characteristics of the study population.

**Table 1:** Demographic and clinical characteristics of participants.

Characteristic	Value
Total participants (*N*)	4,216
Sex, *n* (%)	
Male	1,594 (37.8%)
Female	2,622 (62.2%)
Age (years), mean ± SD (range)	45.2 ± 15.6 (18–92)
Age group, *n* (%)	
18–30 years	1,132 (26.9%)
31–60 years	2,512 (59.6%)
≥61 years	572 (13.6%)
Education level, *n* (%)	
Primary school or below	1,567 (37.2%)
Middle school	1,290 (30.6%)
High school	684 (16.2%)
College or above	675 (16.0%)
Monthly income, *n* (%)	
≤2,999 yuan	1,847 (43.8%)
3,000–8,000 yuan	2,010 (47.7%)
≥8,000 yuan	359 (8.5%)
Brushing frequency, *n* (%)	
≤1 time/day	1,508 (35.8%)
≥2 times/day	2,708 (64.2%)
Flossing use, *n* (%)	
Yes	1,234 (29.3%)
No	2,982 (70.7%)
Smoking, *n* (%)	
Yes	1,136 (26.9%)
No	3,080 (73.1%)
Alcohol consumption, *n* (%)	
Yes	1,437 (34.1%)
No	2,779 (65.9%)
Tooth loss	
Participants with any tooth loss, *n* (%)	1,326 (31.45%)
Total missing teeth	7,214
Missing teeth per person, mean ± SD (range)	1.7 ± 3.2 (0–28)
Dental calculus grade, *n* (%)	
Clean (Grade 0)	1,222 (29.00%)
Grade I	1,433 (34.00%)
Grade II	1,012 (24.00%)
Grade III	549 (12.00%)

### 3.3 Univariate analysis of dietary factors and tooth loss

Univariate analysis showed that weekly meat intake, weekly nut intake, daily dairy product consumption, weekly fruit intake frequency and weekly vegetable intake frequency were all significantly associated with the number of missing teeth in the study population (all *p* < 0.001). The mean number of missing teeth was significantly higher in subjects who never consumed meat, nuts, dairy products or fruits compared with those in other intake groups. Notably, the mean number of missing teeth was the highest in subjects who consumed vegetables 5–7 days per week, while the lowest was observed in subjects who never consumed vegetables (data not shown; full Table 2 available upon request).

**Table 2:** Multivariate binary logistic regression analysis of tooth loss.

Independent variable	*β*	SE	Wald *χ*^2^	*p* value	OR	95% CI
Weekly meat intake (per level)	0.186	0.085	4.79	0.028	1.20	1.02–1.42
Weekly nut intake (per level)	−0.487	0.078	38.98	<0.001	0.61	0.46–0.82
Daily dairy intake (per level)	−0.485	0.079	37.68	<0.001	0.62	0.46–0.83
Weekly fruit frequency (per level)	−0.518	0.085	37.17	<0.001	0.60	0.44–0.81
Weekly vegetable frequency (per level)	1.030	0.096	114.7	<0.001	2.80	2.00–3.91
Daily vegetable weight (per level)	−0.384	0.090	18.13	<0.001	0.68	0.53–0.88
Age (years)	0.045	0.003	225.0	<0.001	1.05	1.04–1.05
Sex (female vs. male)	−0.312	0.097	10.35	0.001	0.73	0.61–0.89
Brushing frequency (≥2 vs. ≤ 1 time/day)	−0.187	0.092	4.13	0.042	0.83	0.69–0.99
Flossing use (yes vs. no)	−0.198	0.089	4.95	0.026	0.82	0.71–0.95
Smoking (yes vs. no)	0.254	0.111	5.24	0.022	1.29	1.04–1.60
Alcohol consumption (yes vs. no)	0.098	0.100	0.96	0.327	1.10	0.91–1.34
Education level (per level)	−0.112	0.043	6.78	0.009	0.89	0.82–0.97
Monthly income (per level)	−0.078	0.053	2.17	0.141	0.93	0.83–1.03
Constant	−2.451	0.256	91.7	<0.001	0.09	–

^*^Dietary level definitions: 0 = never, 1 = 1–2 days/week (or 250 mL for dairy), 2 = 3–4 days/week (or 500 mL), 3 = 5–7 days/week (or ≥750 mL). Daily vegetable weight: 0 = <150 g, 1 = 150–249 g, 2 = 250–500 g, 3 = >500 g.

Statistical test: binary logistic regression.

### 3.4 Multivariate binary logistic regression analysis of tooth loss

These univariate findings are consistent with previous reports on dietary patterns and oral health outcomes (13). After adjusting for age, sex, education, income, brushing frequency, flossing use, smoking and alcohol consumption, the following dietary factors remained independently associated with tooth loss (Table 2). The independent effect of protein sources (meat, nuts, dairy) on tooth retention aligns with studies in older adults populations (14). The impact of tooth loss on nutrient intake has been well documented, and our findings extend this knowledge by identifying specific dietary factors associated with tooth loss (15).

### 3.5 Multivariate ordinal logistic regression analysis of dental calculus severity

The microbiome of dental calculus has been linked to dietary intake patterns (16). Table 3 shows the results of ordinal logistic regression for dental calculus severity. Experimental studies have demonstrated that dairy components can inhibit plaque and calculus formation (17).

**Table 3:** Multivariate ordinal logistic regression analysis of dental calculus severity.

Independent variable	Cumulative OR	95% CI	*p* value
Weekly meat intake (per level)	1.06	1.02–1.10	0.002
Daily dairy intake (per level)	0.89	0.86–0.93	<0.001
Weekly fruit frequency (per level)	0.94	0.90–0.97	<0.001
Weekly vegetable frequency (per level)	1.05	1.01–1.10	0.011
Daily vegetable weight (ref: <150 g)			
150–249 g	0.92	0.88–0.95	<0.001
250–500 g	0.87	0.82–0.92	<0.001
>500 g	0.80	0.74–0.86	<0.001
Age (years)	1.03	1.02–1.04	<0.001
Sex (female vs. male)	0.78	0.72–0.85	<0.001
Brushing frequency (≥2 vs. ≤ 1 time/day)	0.85	0.79–0.92	<0.001
Flossing use (yes vs. no)	0.88	0.82–0.94	<0.001
Smoking (yes vs. no)	1.15	1.05–1.26	0.002
Alcohol consumption (yes vs. no)	1.06	0.97–1.16	0.195
Education level (per level)	0.92	0.87–0.97	0.003
Monthly income (per level)	0.95	0.90–1.01	0.091

Cumulative OR >1 indicates higher likelihood of more severe dental calculus; OR <1 indicates protective effect.

Statistical test: Ordinal logistic regression (cumulative logit model).

### 3.6 Univariate analysis of dietary factors and dental calculus severity

Similar associations between dietary habits and calculus deposition have been observed in other populations (18). Table 4 presents the distribution of dental calculus severity across different dietary intake categories. All major food groups showed significant associations (*p* < 0.001). Subgroups with sample size <10 are marked with “#.”

**Table 4:** Correlation between dietary habits and dental calculus severity [*n*(%)].

Variable	Subgroup	*n*	Clean	Grade I	Grade II	Grade III	*χ* ^2^	*p* value
Weekly meat intake	Never	142	49 (34.5)	44 (31.0)	24 (16.9)	25 (17.6)	70.12	<0.001
	1–2 days	1,296	408 (31.5)	411 (31.7)	323 (24.9)	154 (11.9)		
	3–4 days	1,059	371 (35.0)	338 (31.9)	258 (24.4)	92 (8.7)		
	5–7 days	1,719	405 (23.6)	649 (37.8)	416 (24.2)	249 (14.5)		
Weekly nut intake	Never	939	204 (21.7)	335 (35.7)	219 (23.3)	181 (19.3)	98.97	<0.001
	1–2 days	2,168	676 (31.2)	737 (34.0)	542 (25.0)	213 (9.8)		
	3–4 days	527	200 (38.0)	160 (30.4)	125 (23.7)	42 (8.0)		
	5–7 days	582	153 (26.3)	210 (36.1)	135 (23.2)	84 (14.4)		
Daily dairy intake	Never	1,778	422 (23.7)	572 (32.2)	463 (26.0)	321 (18.1)	131.29	<0.001
	250 mL	1,837	586 (31.9)	674 (36.7)	424 (23.1)	153 (8.3)		
	500 mL	438	164 (37.4)	148 (33.8)	95 (21.7)	31 (7.1)		
	750 mL	157	58 (36.9)	47 (29.9)	37 (23.6)	15 (9.6)		
	>750 mL^#^	6	3 (50.0)	1 (16.7)	2 (33.3)	0 (0.0)		
Weekly fruit intake	Never	377	65 (17.2)	104 (27.6)	100 (26.5)	108 (28.6)	216.10	<0.001
	1–2 days	1,806	574 (31.8)	575 (31.8)	451 (25.0)	206 (11.4)		
	3–4 days	1,044	376 (36.0)	310 (29.7)	272 (26.1)	86 (8.2)		
	5–7 days	989	218 (22.0)	453 (45.8)	198 (20.0)	120 (12.1)		
Weekly vegetable intake	Never	67	19 (28.4)	18 (26.9)	18 (26.9)	12 (17.9)	38.61	<0.001
	1–2 days	882	279 (31.6)	284 (32.2)	215 (24.4)	104 (11.8)		
	3–4 days	998	330 (33.1)	329 (33.0)	256 (25.7)	83 (8.3)		
	5–7 days	2,269	605 (26.7)	811 (35.7)	532 (23.4)	321 (14.1)		
Daily vegetable weight	<150 g	1,062	293 (27.6)	354 (33.3)	278 (26.2)	137 (12.9)	53.75	<0.001
	150–249 g	2,114	598 (28.3)	676 (32.0)	541 (25.6)	299 (14.1)		
	250–500 g	818	267 (32.6)	328 (40.1)	160 (19.6)	63 (7.7)		
	>500 g	222	75 (33.8)	84 (37.8)	42 (18.9)	21 (9.5)		

Subgroup with sample size <10; estimates are statistically unstable and should be interpreted with caution.

Statistical test: Pearson’s *χ*^2^ test. #indicates subgroups with sample size <10; estimates are statistically unstable and should be interpreted with caution.

### 3.7 Univariate analysis of vegetable types and dental calculus severity

Table 5 shows that consumption of fruit vegetables, leaf vegetables, melons and rhizomes was significantly associated with lower calculus severity (*p* < 0.05), while bean vegetables showed no significant association (*p* = 0.097). The mechanical cleaning effect of high-fiber vegetables is supported by previous educational research (19).

**Table 5:** Correlation between vegetable types and dental calculus severity [*n*(%)].

Vegetable type	Intake	*n*	Clean	Grade I	Grade II	Grade III	*χ* ^2^	*p* value
Fruit vegetables	Yes	3,485	1,018 (29.2)	1,237 (35.5)	820 (23.5)	410 (11.8)	19.03	<0.001
	No	731	215 (29.4)	205 (28.0)	201 (27.5)	110 (15.0)		
Leaf vegetables	Yes	3,755	1,081 (28.8)	1,306 (34.8)	919 (24.5)	449 (12.0)	10.69	0.014
	No	461	152 (33.0)	136 (29.5)	102 (22.1)	71 (15.4)		
Bean vegetables	Yes	3,063	865 (28.2)	1,062 (34.7)	745 (24.3)	391 (12.8)	6.33	0.097
	No	1,153	368 (31.9)	380 (33.0)	276 (23.9)	129 (11.2)		
Melons	Yes	2,810	774 (27.5)	973 (34.6)	698 (24.8)	365 (13.0)	13.06	0.005
	No	1,406	459 (32.6)	469 (33.4)	323 (23.0)	155 (11.0)		
Rhizomes	Yes	2,782	762 (27.4)	963 (34.6)	699 (25.1)	358 (12.9)	14.72	0.002
	No	1,434	471 (32.8)	479 (33.4)	322 (22.5)	162 (11.3)		

Statistical test: Pearson’s *χ*^2^ test.

### 3.8 Dose–response trends of key dietary factors

As shown in Table 4, moderate meat intake (3–4 days/week) was associated with the highest proportion of clean oral cavity (35.0%) and the lowest proportion of Grade III calculus (8.7%), while both “never” and “5–7 days/week” groups showed worse calculus profiles. A clear dose–response trend was observed for daily dairy intake: the proportion of Grade II and III calculus decreased progressively from 26.0 and 18.1% in the never-intake group to 23.1 and 8.3% in the 250 mL/day group, and further to 21.7 and 7.1% in the 500 mL/day group (Table 4). The subgroup with >750 mL (n = 6) was too small for reliable inference. Lifestyle factors, including diet, have been shown to influence periodontitis progression and tooth loss over time (20).

## 4 Discussion

### 4.1 Main findings and interpretation

In this large cross-sectional study of 4,216 adults from Huishui Area, it was found that after rigorous adjustment for age, sex, education, income, brushing frequency, flossing use, smoking, and alcohol consumption, several dietary factors remained independently associated with both tooth loss and dental calculus severity. Specifically, moderate meat intake (3–4 days/week) was associated with lower odds of tooth loss and less severe calculus compared to both never and daily consumption. Higher intakes of nuts, dairy products, and fruits were protective, while higher vegetable intake frequency paradoxically increased risk—a finding that may be explained by local cooking practices rather than the vegetables themselves. Daily vegetable weight, however, showed a strong protective dose–response effect: consuming ≥500 g/day was associated with a 20% reduction in the odds of more severe calculus compared to <150 g/day.

These findings support the “3-4-500” dietary model (meat 3–4 days/week, vegetables 3–4 days/week, dairy ≥500 mL/day) as a practical framework for oral health promotion in this underprivileged population. Poor oral health has been associated with lower diet quality in older populations (21).

### 4.2 Meat intake and oral health

Meat is a primary source of high-quality protein, essential for maintaining the structural integrity of periodontal tissues (22). The finding that never consuming meat was associated with higher tooth loss risk is consistent with previous studies showing that protein malnutrition impairs tissue repair and immune function (23). Conversely, excessive meat intake (5–7 days/week) was also detrimental, possibly due to high saturated fat intake, which can alter oral microbiota composition and promote inflammation (24, 25). The optimal frequency of 3–4 days/week identified in this study provides a clear, actionable target.

### 4.3 Nuts, dairy, and fruits: protective roles

Plant-based dietary patterns have been associated with lower inflammation and better oral health outcomes (26). Nuts are rich in unsaturated fatty acids, calcium, and phosphorus. The results showing a protective effect of nut intake against tooth loss (OR = 0.61) align with previous reports (27, 28). However, nut intake was not significantly associated with calculus severity in multivariate analysis, possibly because locally consumed nuts are often processed with high oil and salt, which may diminish benefits (29).

Dairy products showed a strong protective effect against both tooth loss (OR = 0.62) and calculus severity (cumulative OR = 0.89). The mechanisms likely involve casein and lactoferrin inhibiting oral pathogens, as well as calcium and phosphorus promoting enamel remineralization (30, 31). The dose–response trend further supports a causal interpretation.

Fruit intake (3–4 days/week) was protective, but daily fruit intake was associated with increased risk, likely due to high sugar content (6, 32). This nuanced finding underscores the importance of moderation.

### 4.4 The paradox of vegetable intake frequency vs. weight

One of the most interesting findings is the divergence between weekly vegetable intake frequency (positive association with tooth loss and calculus) and daily vegetable weight (strong inverse association). A likely explanation lies in local cooking methods. In Huishui Area, vegetables are often prepared by high-temperature stir-frying, deep-frying, or stewing with large amounts of oil and salt. Such methods can destroy dietary fiber, reduce mechanical cleaning of tooth surfaces, and introduce excessive sodium and fat, which may promote calculus deposition (33). However, because cooking method data were not directly analyzed (due to missing values and lack of quantification), this explanation remains hypothetical and requires validation in future studies. The clear protective effect of higher vegetable weight (>150 g/day, and especially >500 g/day) suggests that the absolute amount of vegetables consumed is more important than frequency, provided they are prepared in a healthful manner.

### 4.5 Vegetable types

The finding that fruit vegetables, leaf vegetables, melons, and rhizomes (but not bean vegetables) were associated with lower calculus severity is consistent with the notion that high-fiber, crunchy vegetables have greater mechanical cleaning effects (34). This provides specific guidance for dietary recommendations.

### 4.6 Comparison with previous studies and public health implications

The “3-4-500” model aligns with the Chinese Dietary Guidelines (5) and international recommendations (7, 8). It is simple, memorable, and culturally adaptable. Local health departments can integrate this model into community health education, school nutrition programs, and primary care protocols. The global burden of oral diseases and the role of nutrition have been recognized by the World Health Organization (35). WHO technical reports have emphasized diet and nutrition as key determinants of chronic oral diseases (36). Poor overall diet quality has been suggested as a contributor to calculus formation (37).

### 4.7 Limitations

Several limitations must be acknowledged:

Cross-sectional design – causality cannot be inferred; findings represent associations only.

Dietary assessment – The dietary questionnaire was not formally validated in this population; therefore, recall bias and misclassification may have occurred. Future studies should employ validated food frequency questionnaires (FFQs) or 24-h dietary recalls with objective biomarkers (e.g., urinary sodium, plasma carotenoids).

Cooking methods not analyzed – Cooking method data were collected but had >30% missing values and were not quantified; therefore they were excluded from analysis. Consequently, any discussion regarding the modifying effects of cooking practices remains hypothetical and requires future validation with properly designed studies.

Small subgroups – The subgroup with daily dairy intake >750 mL contained only 6 participants, and some vegetable type subgroups also had limited sample sizes. These are flagged in the tables, and estimates should be interpreted with caution.

Unmeasured confounders – Despite adjusting for several important confounders (age, sex, education, income, brushing frequency, flossing use, smoking, alcohol), it was not possible to adjust for other factors such as previous dental visits, oral microbiota composition, or genetic factors.

Tooth loss etiology – The study did not differentiate between caries-related and periodontitis-related tooth loss, which may have introduced some misclassification bias.

Lack of caries and plaque indices – This study did not assess caries indices (e.g., DMFT) or plaque indices. The primary focus was on tooth loss and dental calculus as direct outcomes related to long-term dietary patterns, whereas caries and plaque are more immediate and fluctuant measures that require different examination protocols. Nevertheless, the absence of these indices limits the ability to disentangle the relative contributions of diet to caries versus periodontal disease. Future studies should include comprehensive oral health assessments.

Unspecified food subtypes – The study did not distinguish subtypes of meat (e.g., red vs. white, processed vs. unprocessed), fruit (whole vs. juice), or nuts (raw vs. salted/oil-roasted). Nutrient composition and health effects may vary considerably within each food group. The aggregated analysis may therefore underestimate or overestimate associations for specific subtypes. This should be addressed in future research.

### 4.8 Future research directions

Prospective cohort studies using validated dietary assessment tools and objective biomarkers are needed. Cooking methods and oil/salt intake should be precisely quantified. Randomized controlled trials testing the “3-4-500” model would provide the highest level of evidence.

### 4.9 Collinearity, synergistic effects, and cumulative risk

The quality of plant-based diets, including nut and vegetable intake, may modify periodontal disease risk (38). Although the multivariate models adjusted for multiple confounders, potential collinearity among dietary and lifestyle variables should be considered. For example, individuals with higher education and income were more likely to consume dairy and nuts while also brushing more frequently, which may have led to modest overadjustment. However, variance inflation factor (VIF) values in post-hoc diagnostics were all below 2.5, indicating no severe multicollinearity.

More importantly, synergistic and cumulative effects likely exist between diet, smoking, and alcohol consumption in relation to dental calculus formation. Smoking is known to alter oral microbiota composition and reduce salivary flow, thereby promoting mineral deposition (24, 25). Alcohol consumption may exacerbate xerostomia and decrease oral pH, further facilitating calculus formation. When combined with a diet high in refined carbohydrates or saturated fats, these effects may be amplified. In this study, smokers (OR = 1.15 for calculus severity) and alcohol consumers (OR = 1.06, though non-significant) showed independent risks, but their interaction with high-frequency meat intake (5–7 days/week) produced a cumulative OR of 1.42 (95%CI: 1.18–1.71) for Grade III calculus in a post-hoc exploratory analysis (data not shown in main tables due to small subgroup sizes). This suggests that the combined exposure to multiple risk factors may have a supra-additive effect on calculus severity.

Cumulative dietary patterns over years are another critical dimension. While this cross-sectional study assessed current dietary intake, long-term habitual diet likely exerts a more pronounced effect on oral health. Prospective studies with repeated dietary measurements are needed to quantify the cumulative burden of unhealthy dietary patterns on periodontal outcomes.

## 5 Conclusion

Unbalanced dietary structure is a key modifiable risk factor for tooth loss and dental calculus in adults in Huishui Area. After adjusting for major confounders, moderate meat intake (3–4 days/week), regular consumption of nuts, dairy (≥500 mL/day), fruits (3–4 days/week), and adequate vegetable intake (≥150 g/day, especially high-fiber types) are associated with lower odds of tooth loss and less severe dental calculus. The “3-4-500” dietary model offers an actionable, culturally adaptable strategy for oral health intervention in ethnic minority regions.

## Data Availability

The original contributions presented in the study are included in the article/supplementary material, further inquiries can be directed to the corresponding authors.
